# Synovial sarcoma of the floor of the mouth: a rare case report

**DOI:** 10.1186/s12903-019-0961-8

**Published:** 2020-01-06

**Authors:** Yannan Wang, Feiya Zhu, Kai Wang

**Affiliations:** Department of Oral and Maxillofacial Surgery, The Second Xiangya Hospital of Central South University, Renmin Road, No 139, Changsha, 410011 Hunan China

**Keywords:** Synovial sarcoma, The floor of the mouth, Diagnosis, Surgical treatment, Case report

## Abstract

**Background:**

Head and neck Synovial sarcoma (SS) accounts for 3–10% of all total body SS. It is rare to find it in the oral cavity, especially on the floor of the mouth.

**Case presentation:**

We present a 44-year-old Chinese male, who had been misdiagnosed as fibroadenoma, with a swelling on the right submandibular region for more than 3 months. The radiology examinations and the pathology results indicate the diagnosis of SS of the floor of the mouth.

The patient only had a surgical operation, without radiotherapy and chemotherapy. At the first follow-up, the patient exhibited no clinical or radiographic complications, and the patient was asymptomatic on subsequent visits.

**Conclusions:**

Misdiagnosis results the delay of diagnosis and treatment of SS. Immunohistological analysis might be the most important tool to confirm the diagnosis of SS.

## Background

Synovial sarcoma (SS) is a rare malignant neoplasm of unknown histological origin, accounting for 5.6% ~ 10% of all soft tissue sarcomas [1]. It is generally believed as originating from primitive undifferentiated or pluripotent mesenchymal cells [2].. Most studies have found that the age of patients with SS ranged from 15 to 40 years old [3], and approximately 66% of the patients were male [4].

The tumor usually occurs in close association with tendon sheaths, bursae, and joint capsules, primarily in the para-articular regions of the extremities. Head and neck SS accounts for 3–10% of all total body SS, with high incidences in the hypopharynx, the postpharyngeal region, and the parapharyngeal space [5, 6]. It’s rare to found it in the oral cavity, especially on the floor of the mouth. As we know, only 31 intraoral cases have been reported and 2 of them were on the floor of the mouth [1]. The diagnosis of oral SS is difficult because of atypical clinical features and obscure location. Here we present a case report of SS that was previously misdiagnosed as fibroadenoma.

## Case presentation

A 45-year-old male reported that he had a swelling approximately 5.2 × 2.8 × 5.9 cm on the right submandibular region for more than 2 months and was admitted to the local hospital. At the local hospital, the diagnosis of fibroadenoma on the floor of mouth was made. An incisional biopsy was performed under guidance of ultrasound and was submitted for histopathologic examination, the examination revealed that expansion of the lymphatic vessels could be seen in the right sublingual. After an intra-oral excision in the floor of mouth of the right submandibular region (Inferior to the mylohyoid), the immunohistochemistry showed Vimentin was positive, Ki-67 percentage was about 35%, CD34, S-100, CK, P63 and LCA were negative. After discharged from that hospital, he felt that the swelling had grown again in the same area, even rapidly in the near week.

**Fig. 1:**
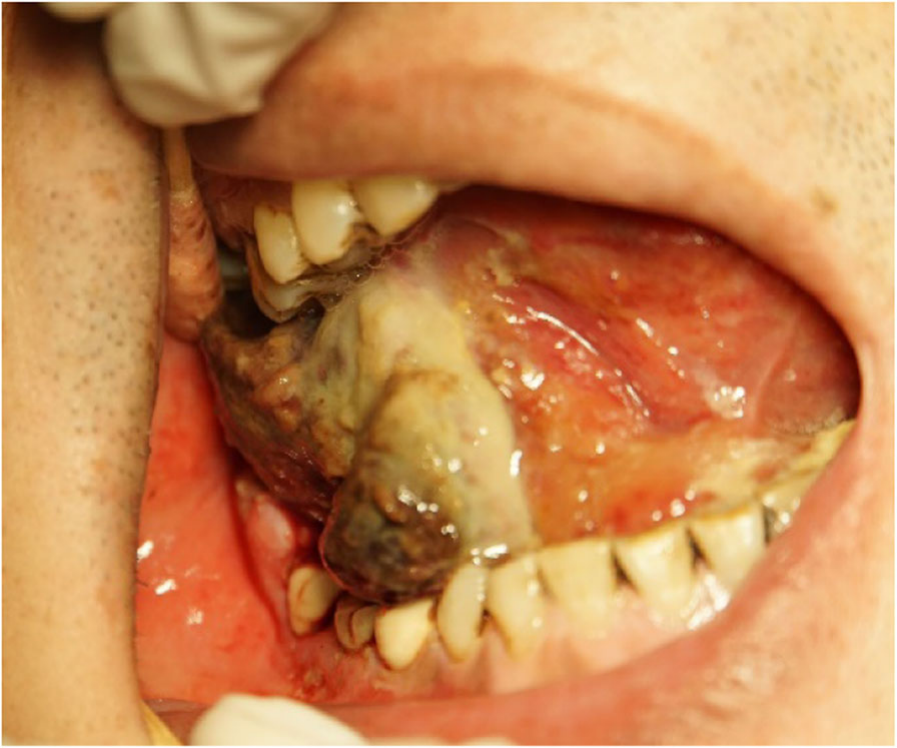
The tumor was located on the right floor of mouth and ventral part of tongue, with bleeding and necrosis on the surface. Due to poor hygiene conditions in the mouth of the patient, there are food residues, etc., and the surface of the tumor is white. The black part is the tumor tissue of ischemic necrosis. Extra oral examination revealed limited mouth opening that was about 1.5 cm

**Fig. 2:**
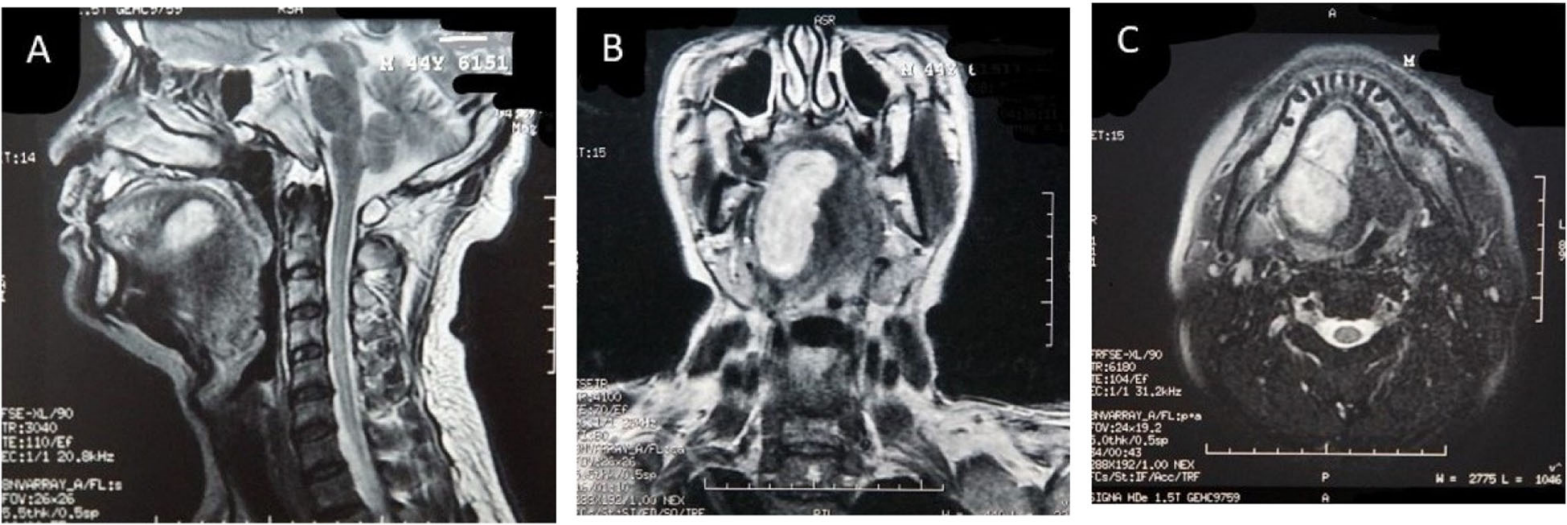
CT examination of different sections of the patient. **a**-**c** There is a clear mass on the right root of tongue and floor of mouth with scattered calcification and liquefaction necrosis

**Fig. 3:**
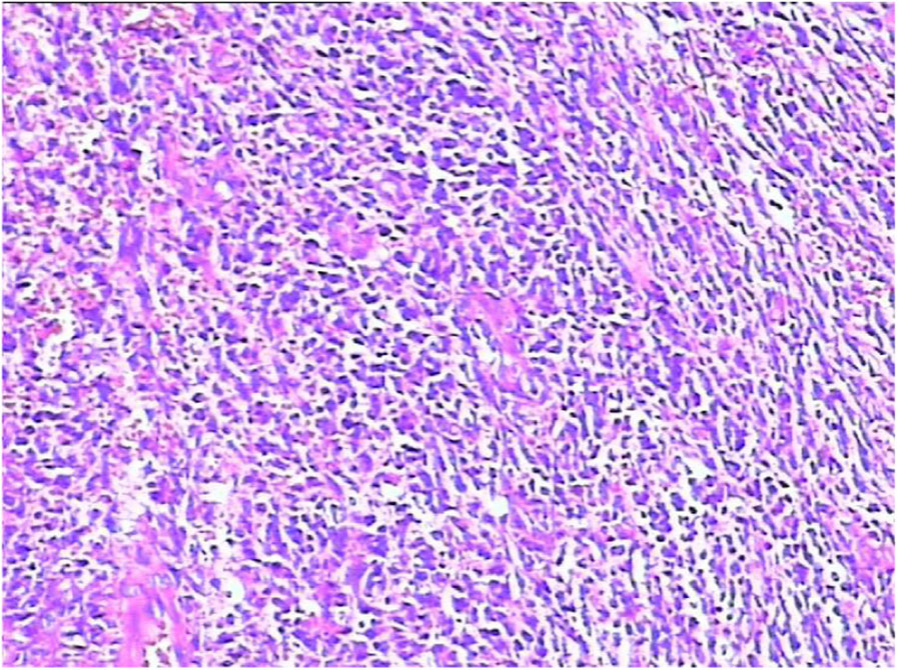
The hematoxylin and eosin (HE)-stained section revealed the tumor composed of spindle cells with a higher proportion of nuclei. HE. × 20

One month after the first operation at local hospital, the patient was referred to our hospital for a growth nodule on the floor of the mouth, associated with pain, numbness, and dyspnea. Intraoral examination revealed a proliferative and ulcerated mass measuring approximately 6.0 × 1.0 cm in the right sublingual involving the right floor of the mouth extending from the alveolus of the left mandibular cuspid to the right mandibular 2nd molar teeth and extending anteriorly crossing the midline of the tongue. The mass was firm, with an unclear border, and the surface covered with black pseudomembranous (Fig. 1). Computed tomography (CT) demonstrated multiple lymph node metastases in the right neck, bilateral submandibular and submental region (Fig. 2a, b), and a mass on right sternocleidomastoid muscle (Fig. 2b, c). Positron Emission Tomography-Computed Tomography (PET-CT) also confirmed the CT demonstration. The hematoxylin and eosin (HE)-stained section revealed the tumor composed of spindle cells with a higher proportion of nuclei, also with indistinct cytoplasmic borders (Fig. 3). The immunohistochemistry revealed epithelial membrane antigen (EMA), CD99, TLE-1, and Bcl-2 were positive while SMA, CD34, CK, CD68, P63, LCA, and S100 were negative. The above results were consistent with a histopthological diagnosis of SS. The patient developed severe dyspnea and lips cyanosis 1 day before the operation and emergency tracheotomy was performed.

**Fig. 4:**
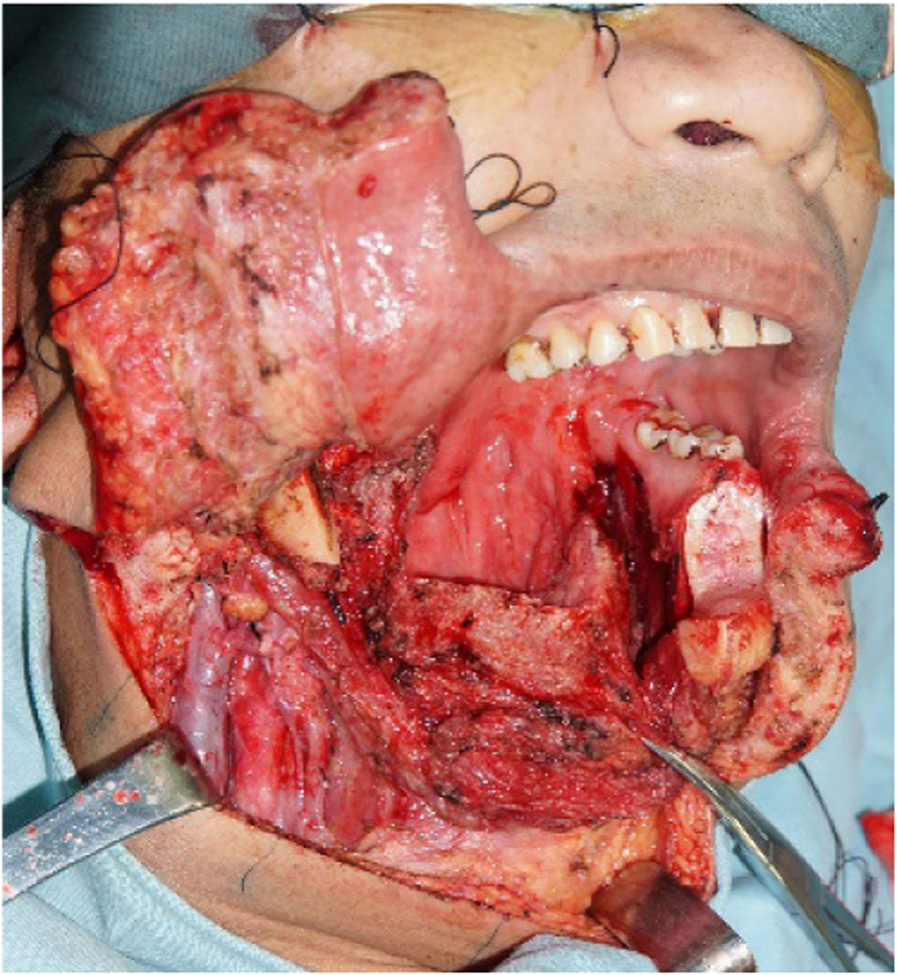
Resection the total tongue and right mandibular

**Fig. 5:**
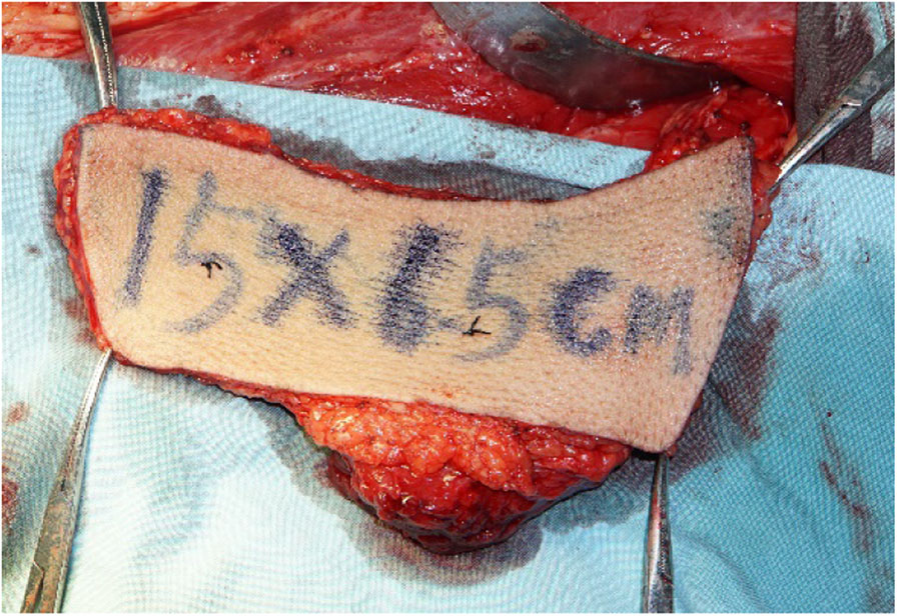
The left thigh anterolateral musculocutaneous flap with some fascia and muscle

**Fig. 6:**
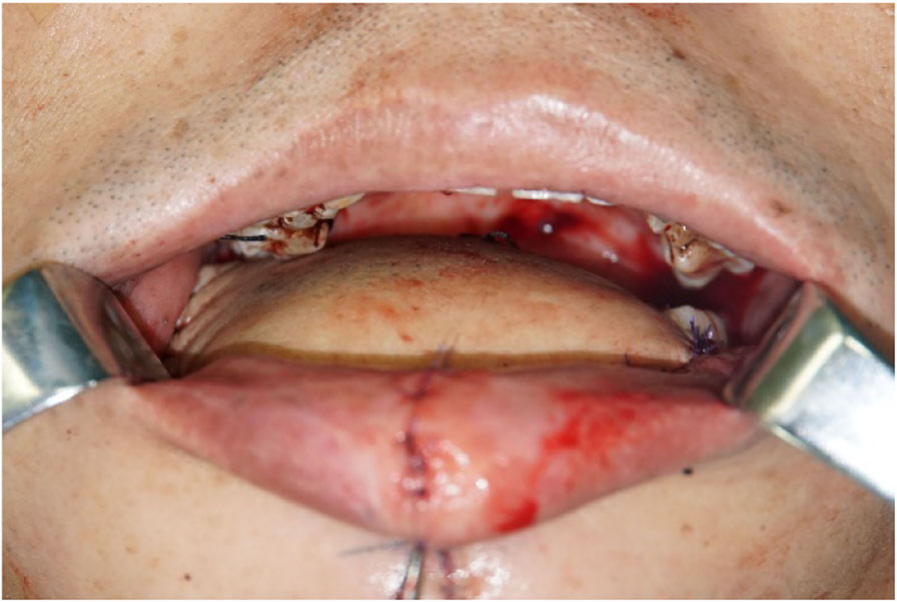
Instant reconstruction of the floor of mouth defect with left thigh anterolateral musculocutaneous flap

**Fig. 7:**
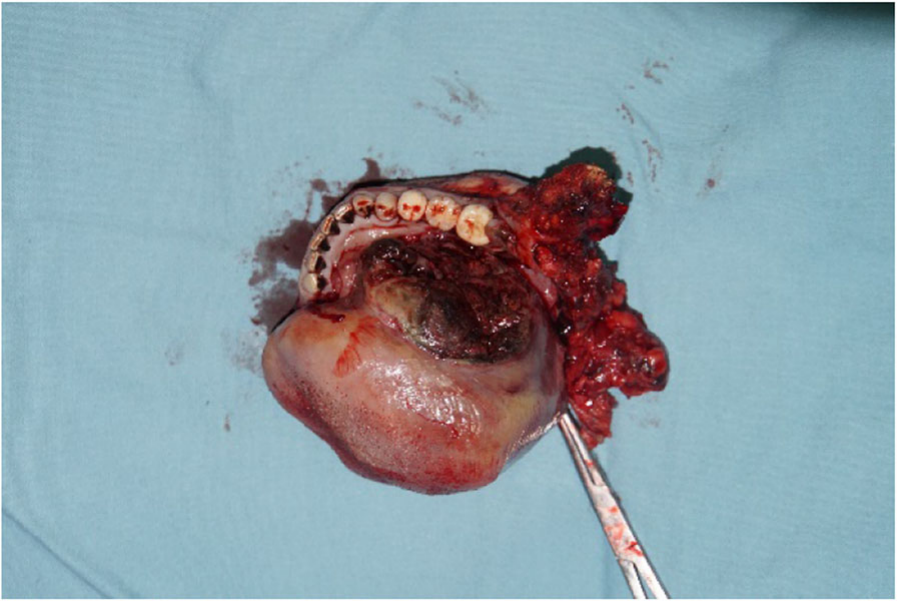
The central part of tumor that was removed showed liquefaction necrosis with some dark brown liquid

The surgical treatment included primary tumor resection, cervical lymph node dissection, and anterolateral musculocutaneous flap reconstruction (Figs. 4, 5, 6, 7), During the operation, about 0.5 × 0.5 × 1 cm tissue was harvested from the anterior, posterior, internal, external, and basal parts of the surgically removed area. The frozen biopsy results indicated complete excision of the tumor. And 9 days after surgery, the patient was discharged.

Within 1 year after surgery, the patient was seen in our out-patient clinic every month to check the recovery and whether there was recurrence or lymph node metastasis. Six months later, the first radiographic postoperative follow-up showed no recurrence. One year after surgery, PET-CT examination was performed, and the patient had no recurrence and distant metastasis. Then the patient was followed up at six monthly intervals and is still alive with no evidence of recurrence after 46 months since undergoing curative intent surgery.

## Discussion and conclusions

### The causes and tissue origin of SS

Synovial sarcoma (SS) was first documented by Simon in 1865 [7] and was so named in 1934 by Sabrazes et al. [8]. The causes and pathogenesis of SS remain unknown. it is generally accepted that SS is derived from mesenchymal stem cells with multiple differentiation potential, and not from synovial tissue [9]. Nearly 90 to 95% of SS demonstrates specific t (x; 18) (p11.2-q11.2) chromosomal translocation that forms the SYT-SSX fusion gene [9–11], which promotes synovial sarcoma cells through the Wnt / β-catenin, PcG, and ERK signaling pathways [12]. Moreover, TGF-β1, Smad, Snail, and Slug are also involved in the development of SS through the EMT pathway [13].

### Clinical manifestations

SS usually occurs in the lower limbs and trunk, especially in the periarticular soft tissues [4]. Head and neck SS accounts for 3–10% of all total body SS [5]. SS lacks typical clinical manifestations, about 50% of presents with pain, some with dysphagia, dyspnea, hoarseness, headache, limited mouth opening, bleeding and lower lip numbness caused by nerve oppression when occurs in the oral cavity [14, 15]. It is usually a slow-growing tumor increasing in size over 1 to 2 years, which is varying from 3 to10 cm [3]. SS could easily be confused with benign tumors in the early stage, as the gradual increase of tumor size, which shows the same symptoms as oral squamous cell carcinoma.

For tumors that have recurred multiple times or diameter > 5 cm, the growth rate tends to accelerate and may cause an emergency. In this case, the patient’s tumor rapidly increased with surface bleeding after admitted to our hospital. Moreover, he even suffered from severe dyspnea due to the tumor and clot blocking the airway. According to the postoperative tumor anatomy, the central part of the tumor showed liquefaction necrosis with some dark brown liquid (Fig. 7), which may infer to be related to the cause of dyspnea. Therefore, physicians should always pay more attention to the patient’s breathing and oxygen saturation for patients with tumor nearing the tongue or soft palate, especially for those with rapid growth or surface bleeding. Preventive tracheostomy may be considered to prevent suffocation.

### Diagnosis and differential diagnosis

In our case, the patient was previously diagnosed as fibroadenoma because of the rarity of SS and lack of typical clinical and imaging manifestations. Although Pantomography, CT, MRI, and PET-CT can be used as diagnostic tools, smaller SSs often show similar imaging features to benign tumors on CT and MR imaging [16, 17]. Despite the lack of specific imaging findings, CT and MRI are still useful for determining the location of the primary tumor, adjacent tissue infiltration, and metastasis.

Synovial sarcoma can be classified into three major histopathological subtypes: 1) monophasic SS containing uniform spindle cells or epithelial cells, 2) biphasic SS composed of epithelial cells arranged into glandular structures with spindle cells arranged into fascicles, 3) poorly differentiated SS characterized by the presence of spindle and/or round blue cells [18]. In histology, synovial sarcoma needs to be differentiated from metastatic adenocarcinoma, malignant fibrous histiocytoma (MFH) and fibrosarcoma. However, due to the diversity of morphological manifestations, the pathological diagnoses of atypical cases are very difficult [19].

The other cause of misdiagnosis might be the previous immunohistochemistry lacked some key immune marker detection such as EMA, CD99, TLE-1, and Bcl-2, etc. So far, there is no single immunological marker specific to synovial sarcoma has been found. SS shows positive of TLE-1, AE1 / AE3, EMA, CK7, CK19, Vimentin, Bcl-2, CD99, and S-100 while generally shows negative express of CD34, CD31, actin (HHF-35) or Myoglobin [20, 21]. Some studies reported that TLE-1 expression, a sensitive and specific marker for SS, could be as high as 90%, and Bcl-2 expression rate was 93%, CD99 was 73%, While S100 was locally expressed in 21% of cases [22–24].

For some cases with atypical histological morphology or confusing results of immunohistochemistry, detection of SYT-SSX fusion gene by molecular biology or cytogenetic technology can be used to help diagnose with synovial sarcoma [20]. It was said that nearly 90 to 95% of SS demonstrated specific t (x; 18) (p11.2-q11.2) chromosomal translocation that forms the fusion gene SYT-SSX [9–11]. The detection rate of FISH was about 80% and RT-PCR was 83.8%, and the combined detection rate of both was 92.9% [25].

### Treatment and prognosis

The optimal approach to the treatment of SS is still unclear and there is no standard treatment protocol [6]. It is recommended wide-local excision [20], and adjuvant radiation with or without chemotherapy [9]. At present, for synovial sarcoma in head and neck, simple surgical treatment can be recommended for smaller and superficial lesions; but for the larger and deeper SS, surgery and radiotherapy combined treatment can be considered since it is difficult to perform completely extended resection as many nerve and vessels involved in the head and neck region. The defects after resection can be repaired with flap transfer [26].

In our case, chemotherapy was not performed. The importance of chemotherapy has not been widely acknowledged due to the lack of evidence that chemotherapy was associated with improved overall survival. According to the Yang’s report [27], after observation of 21 patients with synovial sarcoma of the head and neck, it was found that postoperative chemotherapy slightly prolonged the time for the occurrence of distant metastasis but showed no significant difference for the overall survival rate or local recurrence. On the other hand, radiotherapy for oral tumors sometimes leads to adverse effects such as radiation mucositis, ulceration, and osteoradionecrosis [1]. According to Zhou’s report [28], for soft tissue sarcoma of the maxillofacial region, the five-year survival rate of those who underwent preventive cervical lymph node dissection was higher than those who did not undergo cervical lymph node dissection.

The prognosis of synovial sarcoma is often associated with tumor location, size, patients’ age, surgical procedure, degree of differentiation [29]. Generally believed that: 1) age < 60 years old, 2) total tumor dimension < 5 cm, 3) extensive calcification, 4) appropriate surgical resection, 5) a high degree of tumor differentiation, extensive hemorrhagic necrosis and high mitosis index, 6) tumor without distant metastasis, will lead to good prognosis [30–32]. However, due to the insidious onset, patients see doctors always because of discomfort or dysfunction with large tumors, and the curative effect is often poor, the recurrence or metastasis generally occurs in the first two years after the initial treatment [1]. The recurrence rate is 20.8% and the metastatic rate is 29.2%, the most common metastatic sites are lung, followed by local lymph nodes and bone [1, 30–32]. The three-year survival rate is about 50% [33].

In this case, as the rarity SS tumor, the patient was misdiagnosed at the local hospital. Immunohistological analysis might be the most important tool to confirm the diagnosis of SS. Surgical treatment focus on primary tumor resection without chemotherapy might be considered according to the patient’s situation.

## Data Availability

The complete data and materials described in the case report are not publicly available due anonymity purposes but are available from the Department of Oral and Maxillofacial Surgery, The Second Xiangya Hospital of Central South University on reasonable request.

## Abbreviations

CT: Computed tomography
EMA: Epithelial membrane antigen
HE: The hematoxylin and eosin
MFH: Malignant fibrous histiocytoma
PET-CT: Positron Emission Tomography-Computed Tomography
RT-PCR: Reverse Transcription-Polymerase Chain Reaction
SS: Synovial sarcoma
